# THE ROLE OF AMBIVALENCE ON WELL-BEING OF AGING PARENTS WHO HAVE A DISABLED CHILD: MULTILEVEL MEDIATION APPROACH

**DOI:** 10.1093/geroni/igz038.146

**Published:** 2019-11-08

**Authors:** Eun Ha Namkung

**Affiliations:** Brandeis University, Waltham, Massachusetts, United States

## Abstract

According to the family systems theory, strains from parenting an adult with disabilities may spillover to parents’ relationships with their other children and disrupt family dynamics and their well-being in later life. This study examined whether parental ambivalence toward their non-disabled children is greater in families of adults with disabilities [developmental disabilities (DD) or serious mental illnesses (SMI)] than families without an adult child with disabilities. The study also investigated whether ambivalence mediates the associations of having an adult child with DD or SMI on parents’ health. Data were from the 2011 Wisconsin Longitudinal Study in which aging parents (Mage = 71; n = 6,084) were asked about their relationship with each of their adult children. Multilevel regression models and multilevel structural equation models (MSEM) were estimated to analyze the data. Our findings showed that parents of an adult with SMI felt greater ambivalence toward their non-disabled adult children than comparison group parents of adults without disabilities, whereas no significant differences were found between parents of an adult with DD and comparison group parents. Parental ambivalence toward their non-disabled adult children played a significant indirect role in the negative association between having a child with SMI and parental physical and mental health, after adjusting for parent- and child-characteristics associated with parental health and/or ambivalence. The findings have implications for clinical practice with aging families of adults with disabilities and suggest the need for additional research to better understand intergenerational dynamics in these families.

